# Human-derived acellular dermal matrix may be an alternative to autologous grafts in tympanic membrane reconstruction: systematic review and meta-analysis

**DOI:** 10.1186/s40463-021-00518-w

**Published:** 2021-07-06

**Authors:** Shan Xu, Xia Sun, Ning Yang, Aihui Yan

**Affiliations:** Department of Otolaryngology, The First Hospital of China Medical University, Shenyang, Liaoning 110001 People’s Republic of China

**Keywords:** Acellular dermal matrix, Autologous grafts, Tympanoplasty

## Abstract

**Background:**

Human-derived acellular dermal matrix (ADM) has been widely used as an effective alternative to autologous grafts in tympanoplasty. However, evidence of ADM as an alternative to autologous grafts in the repair of tympanic membrane (TM) perforation still lacks adequate empirical evidence.

**Objectives:**

To determine the clinical safety and efficacy of human-derived ADM as TM graft material for tympanoplasty.

**Data sources:**

The PubMed, EMBASE, Cochrane Library, EBSCO, Ovid, Scopus, and Web of Science databases and reference lists of the retrieved articles were searched, with no language restriction.

**Selection criteria:**

All randomized controlled trials and retrospective cohort studies that compared the use of human-derived ADM and autologous grafts in tympanoplasty for TM perforation were included.

**Data collection and analysis:**

Two review authors independently assessed risk of bias in the included studies and extracted data. The pooled results for continuous data were reported as a mean difference (MD) and 95% confidence intervals (CI). For dichotomous data, odds risk (OR) with 95% CI was used. ChI^2^ statistic and Galbraith plots were used to assess the heterogeneity. Publication bias was assessed with a funnel plot and Egger’s test.

**Main results:**

Five retrospective cohort studies and four randomized controlled studies with a total of 610 participants were included in the meta-analysis. No significant differences in graft success (OR: 0.71 [0.39, 1.29], *p* = 0.26), air-bone gap (ABG) reduction (MD: − 0.59 [− 3.81, 1.19], *p* = 0.51), or complications (OR: 1.23 [0.07, 20.64], *p* = 0.89) were found between the ADM group and autologous graft group. The use of ADM significantly shortened tympanoplasty surgery time (MD: − 16.14 [− 21.22, − 11.07], *p* < 0.00001) and reduced postoperative pain (MD: − 2.57 [− 3.57, − 1.58], *p* < 0.00001) compared with the autologous graft group.

**Conclusion:**

Human-derived ADM might be an effective alternative to autologous grafts for tympanoplasty. However, some of the studies that were included in the present meta-analysis had rather low methodological quality, and more adequately designed clinical trials should be performed in the future.

**Graphical abstract:**

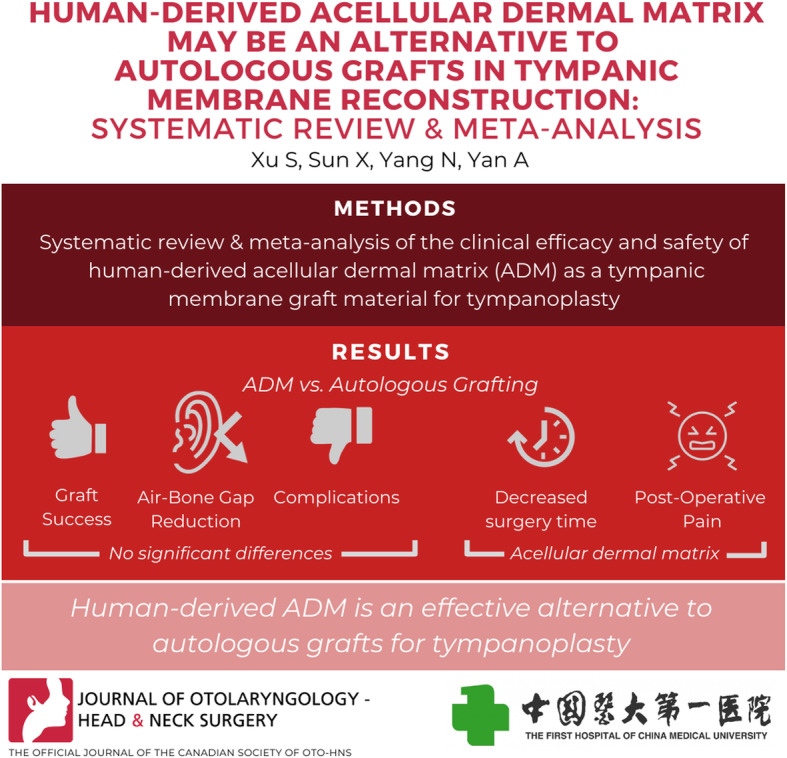

**Supplementary Information:**

The online version contains supplementary material available at 10.1186/s40463-021-00518-w.

## Background

Tympanic membrane (TM) perforation is a common health issue in otolaryngology. Tympanic membrane perforation is usually caused by trauma, chronic otitis media, or surgical complications and presents with conductive hearing loss [1]. Although most TM perforations heal spontaneously, some patients require surgery [2].

Tympanoplasty involves surgical repair of the TM. Tympanoplasty was introduced in the 1950s [3]. Different types of graft materials have been used to reconstruct the TM, including temporalis fascia, cartilage perichondrium, adipose tissue, muscle fascia, and scar tissue [4–7]. The temporalis fascia and tragal perichondrium are the most commonly used graft materials, with a good success rate for TM reconstruction [8–11]. However, additional incisions are required during surgery to harvest these materials, which consequently increases patient suffering and the risk of postoperative infection.

Acellular dermal matrix (ADM) is a soft tissue graft that is created by the decellularization of tissue, leaving the extracellular matrix. The dermal surface of ADM is favorable for the growth of epithelial cells and vascularization, which can help achieve rapid epithelialization [12]. As cellular components of the epidermis and dermis are removed, ADM can be tolerated in the host without triggering an immune response. Additionally, ADM is produced under aseptic conditions to ensure that the allograft is sterile and free of viruses, bacteria, and spores. A cellular dermal matrix has long been used as a soft tissue replacement, and it is commonly used in the field of wound healing, tissue repair, and reconstruction [13]. In recent years, ADM has also been widely used as graft material in tympanoplasty [14].

Animal studies showed that ADM exhibits excellent closure rates in repairing induced TM perforations compared with autologous grafts [15–18]. Recent clinical trials have also demonstrated the efficacy of ADM in the repair of TM perforations. The use of ADM to repair TM perforations is promising because it is readily available and relatively easy to apply [19–21]. Acellular dermal matrix was shown to be an effective alternative to autologous grafts in some randomized controlled trials [22–25]. Retrospective studies also confirmed these success rates, and hearing improvements were similar for ADM and autologous grafts for tympanoplasty [8, 26–29].

However, evidence of ADM as an alternative to autologous grafts in the repair of tympanic membrane (TM) perforations still lacks adequate empirical evidence. Therefore, we performed a meta-analysis of comparative studies to determine the clinical safety and efficacy of ADM as TM graft material for tympanoplasty.

## Methods

This meta-analysis was performed according to Preferred Reporting Items for Systematic Reviews and Meta-Analyses (PRISMA) guidelines for meta-analyses of intervention trials.

### Types of studies

Randomized controlled trials and retrospective cohort studies that compared the use of ADM and autologous grafts in tympanoplasty for TM perforations were included in the present meta-analysis. The included studies had to be published as full papers. Studies that reported results only as abstracts were not included.

### Types of participants

Patients who underwent tympanoplasty for TM perforations that were caused by trauma, chronic otitis media, or middle ear cholesteatoma were included in the meta-analysis.

### Types of interventions

Interventions of interest included those that used ADM or autologous grafts, such as temporalis fascia, tragal perichondrium, and mastoid process periosteum, for TM grafting in tympanoplasty.

### Inclusion and exclusion criteria

The outcomes included graft success rate, surgery time, postoperative pain, hearing gain, and complications. The inclusion criteria were (1) randomized controlled trials or retrospective cohort studies, (2) only adult patients, and (3) patients who underwent type I tympanoplasty. The exclusion criteria included (1) pediatric patients, (2) patients who underwent ossicular chain reconstruction, and (3) patients who underwent type 3 tympanoplasties or other ossicular procedures.

### Outcome and measures

Graft success was defined as closure of the TM perforation within the follow-up period. Surgery time was defined as the interval between the time of ear speculum insertion into the ear canal and time of postoperative dressing application. Postoperative pain was measured using a 10 cm visual analog scale (VAS), which consisted of a 10 cm long horizontal line, marked from 0 on the left to 10 on the right, with 0 representing no pain and 10 denoting the highest possible pain intensity. This VAS has been demonstrated to be a reliable method of self-rating pain intensity. Audiometric outcomes were measured using pure-tone auditory tests, air conduction (AC) thresholds, bone conduction (BC) thresholds, and air-bone gap (ABG) to evaluate hearing gain. Complications were defined as all adverse events that were related to the intervention that occurred during the follow-up period.

### Data sources and search strategy

We searched the PubMed, EMBASE, Cochrane Library, EBSCO, Ovid, Scopus, and Web of Science databases for all articles since the databases’ inception to October 2020. We also searched the retrieved articles’ reference lists, with no language restriction. The searches were conducted using controlled search terms. We searched both titles and abstracts. The search terms were the following: (acellular dermis OR acellular dermal tissue OR acellular dermal graft tissue OR decellularized dermal scaffold OR acellular dermal matrix) AND (tympanoplasty OR tympanoplasties). A full description of the search strategy is presented in Additional File 1.

### Selection of studies

Two authors of the present meta-analysis independently read the titles and abstracts of the articles that were retrieved based on the search terms. Two authors independently retrieved and evaluated the full text of the potentially relevant studies. Any disagreements among the authors about the eligibility of articles were discussed and resolved collectively to determine the articles’ ultimate eligibility for inclusion in the analysis.

### Data extraction and management

We prepared a data extraction form for data extraction. Two authors extracted the data, and any differences in the extracted data among the authors’ completed forms were subsequently reviewed by a third author until agreement was reached.

### Risk of bias assessment

Methodological quality was assessed by two authors independently. The risk of bias of the randomized controlled trials was assessed using the Cochrane risk of bias tool. This tool consists of domains of sequence generation, allocation concealment, blinding of participants and personnel, blinding of outcome assessors, incomplete outcome data, selective reporting, and other sources of bias. Each domain was rated as low, unclear, or high risk. Non-randomized controlled trials were assessed using the Newcastle-Ottawa Quality Assessment Scale. This scale consists of domains of selection, comparability, and outcome. It identifies “high” quality choices with stars for every domain, with a maximum of eight stars per study.

### Statistical analysis

We performed the data synthesis and statistical analysis using Review Manager software. For continuous data, we used the mean and standard deviation (SD) that were reported in the original study. Pooled results for continuous data are reported as the mean difference (MD) and 95% confidence interval (CI). For dichotomous data, we used odds risk (ORs) and 95% CI.

ChI^2^ statistic was used to assess heterogeneity among trials. We regarded heterogeneity as substantial if I^2^ > 50%. Galbraith plots were used to explore potential sources of heterogeneity. If clinical heterogeneity was found, then pooled outcomes were calculated using random-effects meta-analysis. For all other analyses, fixed-effect meta-analysis was used for combining data.

Because we included randomized controlled trials and retrospective cohort studies, we conducted a subgroup analysis according to the trial design to reduce the heterogeneity of all outcomes. For important outcomes, we performed sensitivity analyses to explore the effect of risk of bias by temporarily excluding studies with a high risk of bias to determine whether this impacted the results. Publication bias was assessed by funnel plots and Egger’s test. Egger’s linear regression test was used to evaluate asymmetry. Values of *p* < 0.05 were considered statistically significant.

## Results

### Included studies

**Fig. 1:**
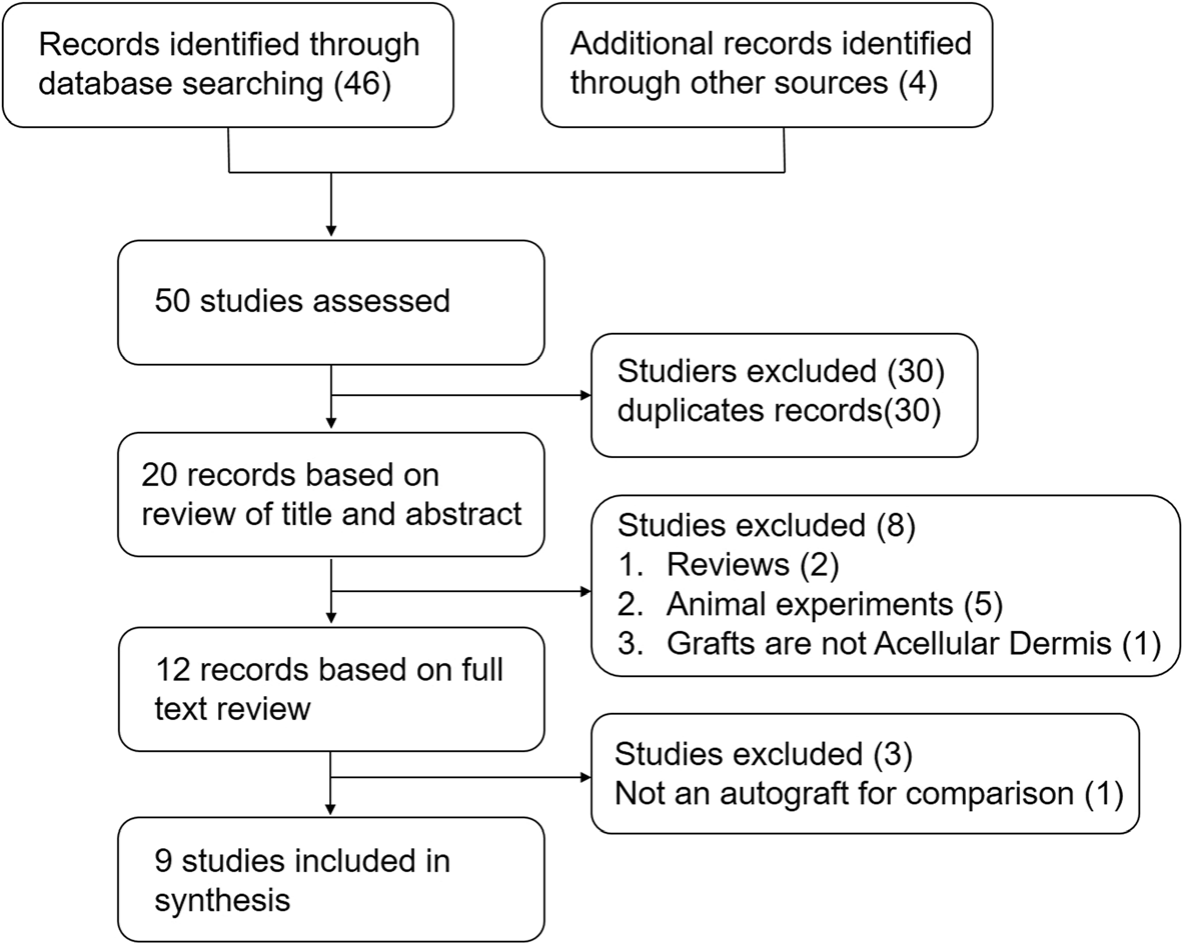
Results of literature review

The electronic search initially retrieved 50 articles, 20 of which were retained after removing duplicates. After screening the titles and abstracts, the search identified 12 possibly relevant studies [8, 19–29]. Based on a review of the full text, three clinical studies were excluded. Two studies did not use autologous grafts as a control group [19, 21], and one study did not focus on TM perforation [20]. Five retrospective cohort studies and four randomized controlled studies with a total of 610 participants were included in the present meta-analysis [8, 22–29]. For further details, see Fig. 1.

All of the included studies used human-derived ADM for TM grafting. Four studies used the temporalis fascia as the control group [8, 23, 24, 29], three used the tragal perichondrium [22, 26, 28], and two combined multiple autologous grafts as the control group [25, 27]. Three studies only included patients with suppurative otitis media [24, 25, 29], and one study included patients with middle ear cholesteatoma [23]. ,The other studies did not report causes of the participants’ TM perforation. One study did not report the length of the follow-up period [27], For the other studies, the follow-up length was 3–24 months. Characteristics of the included studies and participants are reported in Tables 1 and 2.

**Table 1:** Characteristics of the include studies

Study	Design	Participants	Interventions		Follow-up	Outcomes	Primary Results
			Experimental	Control			
Benecke 2001 [8]	Retrospective cohort study	Patients underwent tympanoplasty	ADM (human-derived), *n* = 20	Autologous temporalis fascia, n = 20	6 months	Graft success rate; Audiometric tests(ABG)	ADM is a suitable material for TM grafting
Min 2018 [26]	Retrospective cohort study	Patients with tympanic membrane perforation who underwent type I tympanoplasty	ADM (human-derived), *n* = 26	Autologous tragal perichondrium, *n* = 27	3 months	Graft success rate; Operation time; Postoperative pain; Audiometric tests(ABG,BC)	Tympanoplasty using ADM can be achieved with similar postoperative results and less pain.
Vos 2005 [27]	Retrospective cohort study	Patients underwent type I tympanoplasty without mastoidectomy or ossicular chain reconstruction	ADM (human-derived), *n* = 25	Autologous temporalis fascia, *n* = 56; Autologous fascia plus cartilage reconstruction, *n* = 33	Not report	Graft success rate; Audiometric tests(ABG); Complications	ADM is an effective TM graft when used in type I tympanoplasty
Yang 2019 [28]	Retrospective cohort study	Patients of tympanic membrane perforations who underwent tympanoplasty	ADM (human-derived), n = 27	Autologous tragal perichondrium, *n* = 34	6 months	Graft success rate; Operation time; Audiometric tests(ABG)	ADM can be recommended as an attractive alternative to cartilage grafts
Fu 2017 [29]	Retrospective cohort study	Chronic suppurative otitis media patients underwent type I tympanoplasty	ADM (human-derived), *n* = 32	Autologous temporalis fascia, *n* = 28	3 months	Graft success rate; Operation time; Postoperative pain Audiometric tests(ABG)	The effect of ADM for repairing tympanic membrane is similar to the temporalis fascia.
Lee 2018 [22]	Prospective randomised controlled study	Patients who underwent type I tympanoplasty for tympanic membrane perforation	ADM (human-derived), n = 27	Autologous tragal perichondrium, n = 33	6 months	Graft success rate; Operation time; Postoperative pain; Audiometric tests(ABG,AC,BC); Complications	ADM was shown to be an effective alternative to tragal perichondrium.
Li 2014 [23]	Randomised controlled study	Middle ear cholesteatoma patients underwent tympanoplasty for tympanic membrane perforation	ADM (human-derived), *n* = 30	Autologous temporalis fascia, *n* = 22	12 months	Graft success rate; Audiometric tests(ABG)	ADM was shown to be an effective alternative to the temporalis fascia
Raj 2011 [24]	Prospective randomized unblinded controlled trial	chronic suppurative otitis media patients underwent type I tympanoplasty	ADM (human-derived), n = 20	Autologous temporalis fascia, n = 20	3 months	Graft success rate; Operation time; Postoperative pain; Audiometric tests(ABG,BC)	ADM as graft material are comparable to temporalis fascia in terms of graft uptake and hearing improvement.
Liao 2017 [25]	Randomised controlled study	Chronic suppurative otitis media patients undergoing tympanoplasty	ADM (human-derived), *n* = 18	Tragus cartilage-perichondrium, *n* = 69; Mastoid process periosteum, *n* = 21; Temporalis fascia, n = 28	24 months	Graft success rate; Operation time; Audiometric tests(ABG)	ADM is suitable for small and medium-sized perforation.

*TM* Tympanic membrane; *ADM* Acellular dermal matrix; *AC* Air conduction *BC* Bone conduction *ABG* Air-bone gap

**Table 2:** Characteristics of participants

Study	Country	n	Average age	Causes of TM perforation	Mean perforation size%	ADM derivation	Autologous grafts	Type of surgery	Anesthesia
Benecke 2001 [8]	USA	40	NR	NR	NR	human	Temporalis fascia	Type 1 tympanoplasty	NR
Min 2018 [26]	Korea	53	53.2	NR	22.90%	human	Tragal perichondrium	Type 1 tympanoplasty	Local anesthesia
Vos 2005 [27]	USA	108	20	NR	45%	human	Multiple grafts	Type 1 tympanoplasty	General anesthesia
Yang 2019 [28]	China	61	29.2	NR	NR	human	Tragal perichondrium	Type 1 tympanoplasty	General anesthesia
Fu 2017 [29]	China	60	31.5	Chronic suppurative otitis media	NR	human	Temporalis fascia	Type 1 tympanoplasty	Local anesthesia
Lee 2018 [22]	Korea	60	53.3	NR	NR	human	Tragal perichondrium	Type 1 tympanoplasty	Local anesthesia
Li 2014 [23]	China	52	53	Middle ear cholesteatoma	NR	human	Temporalis fascia	Type 1 tympanoplasty	General anesthesia
Raj 2011 [24]	India	40	NR	Chronic suppurative otitis media	NR	human	Temporalis fascia	Type 1 tympanoplasty	Local anesthesia
Liao 2017 [25]	China	136	41	Chronic suppurative otitis media	NR	human	Multiple grafts	Type 1 tympanoplasty	NR

*TM* Tympanic membrane; *ADM* Acellular dermal matrix; *NR* Not report

### Risk of bias

For retrospective cohort studies, we assessed the risk of bias in terms of case selection, comparability, and the adequacy of outcome. The reporting quality of two studies was determined to be adequate [26, 28]. One study was assessed as having a high risk of case selection bias because of no description of derivation of the cohort was provided [8]. Two studies were assessed as having a high risk of comparability bias because the authors only reported one factor of the participants and no additional factor for the study design control [8, 29]. Two studies were assessed as having a high risk of adequacy of outcome bias (one study because no details of the assessment of outcome were reported [8], and one study because it did not report the length of the follow-up period [27]). Further details of the risk of assessment bias for the retrospective cohort studies are reported in Table 3.

**Table 3:** Risk of bias assessment of retrospective cohort studies

stduy	Selection				Comparability	Outcome		
	Item 1	Item 2	Item 3	Item 4	Item 5	Item 6	Item 7	Item 8
Benecke 2001 [8]		☆	☆	☆	☆		☆	☆
Min 2018 [26]	☆	☆	☆	☆	☆☆	☆	☆	☆
Vos 2005 [27]	☆	☆	☆	☆	☆☆	☆		
Yang 2019 [28]	☆	☆	☆	☆	☆☆	☆	☆	☆
Fu 2017 [29]	☆	☆	☆	☆	☆	☆	☆	☆

Item 1: Representativeness of the exposed cohort

Item 2: Selection of the non - exposed cohort

Item 3: Ascertainment of exposure

Item 4: Demonstration that outcome of interest was not present at start of study

Item 5: Comparability of cohorts on the basis of the design or analysis

Item 6: Assessment of outcome

Item 7: Was follow-up long enough for outcomes to occur

Item 8: Adequacy of follow up of cohorts

For randomized controlled studies, we assessed the risk of bias in terms of allocation sequence generation, blinding, the incomplete reporting of outcome data, and selective reporting. Overall, the methodological quality of the randomized controlled studies was inadequate. None of the included randomized controlled studies adequately provided methodological information. Only one study reported the method that was used to generate the random sequence [22]. One study was assessed as having a high risk of performance bias because it was an unblinded controlled trial [24]. However, all of the studies reported complete outcome data. A summary of methodological quality for the randomized controlled studies is provided in Table 4.

**Table 4:** Risk of bias assessment of randomised controlled studies

stduy	Selection bias		Performance bias	Detection bias	Attrition bias	Reporting bias	Other bias
	Item 1	Item 2	Item 3	Item 4	Item 5	Item 6	Item 7
Lee 2018 [22]	Low risk	Unclear risk	Unclear risk	Unclear risk	Low risk	Unclear risk	Unclear risk
Li 2014 [23]	Unclear risk	Unclear risk	Unclear risk	Unclear risk	Low risk	Unclear risk	Unclear risk
Raj 2011 [24]	Unclear risk	Unclear risk	High risk	High risk	Low risk	Unclear risk	Unclear risk
Liao 2017 [25]	Unclear risk	Unclear risk	Unclear risk	Unclear risk	Low risk	Unclear risk	Unclear risk

Item 1: Random sequence generation;

Item 2: Allocation concealment;

Item 3: Blinding of participants and personnel;

Item 4: Blinding of outcome assessment;

Item 5: Incomplete outcome data;

Item 6: Selective reporting;

Item 7: Bias due to problems not covered by [1] to [6] above

#### Heterogeneity assessment

**Fig. 2:**
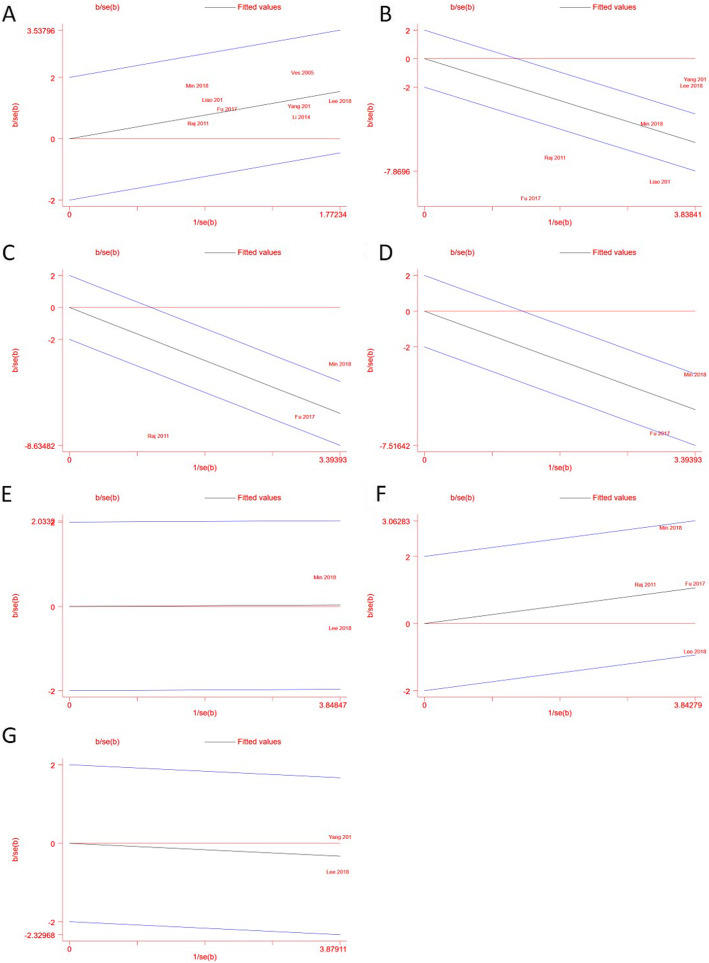
Galbraith plots of outcomes. Heterogeneity assessment. Galbraith plots of pooled outcomes, study plots outside the 95% confidence interval indicate heterogeneity. (**a**) Graft success rate; (**b**) Operation time; (**c**) Immediate postoperative pain, study Raj 2011included; (**d**) Immediate postoperative pain, study Raj 2011excluded; (**e**) Bone conduction; (**f**) Air-bone gap; (**g**) Air-bone gap reduction. The outcomes of pain one day after surgery and air conduction are unavailable due to insufficient number of studies

ChI^2^ statistic was used to assess heterogeneity. We regarded heterogeneity as substantial if I^2^ > 50%. Galbraith plots were used to explore potential sources of heterogeneity. Galbraith plots provides a graphical display to get a visual impression of the amount of heterogeneity from a meta-analysis. For each study is plotted according z statistic, and the regression line constrained through the origin, with its 95% confidence interval, when the plots of studies out of the confidence bounds, indicate the studies may be the source of heterogeneity. According to the assessment, there is heterogeneity in the outcomes of surgery time and immediately postsurgery pain, and there is a low risk of heterogeneity in other outcomes. Further details of the heterogeneity assessment reported in Fig. 2 and Table 5.

**Table 5:** Heterogeneity and publication bias assessment

Outcomes	I^2^	Potential source of heterogeneity	Egger’s test
Graft success rate	0%	none	*p* = 0.835
Surgery time	90%	Fu 2017 [29]; Yang 2019 [28]; Lee 2018 [22]; Liao 2017 [25]; Raj 2011 [24]	*p* = 0.016
Postoperative pain (immediately postsurgery)	91%	Raj 2011 [24]	*p* = 0.178
Postoperative pain (1 day postsurgery)	Not applicable	Not applicable	Not applicable
Hearing results (average AC)	Not applicable	Not applicable	Not applicable
Hearing results (average BC)	0%	none	Not applicable
Hearing results (average ABG)	38%	none	*p* = 0.522
Hearing results (ABG reduction)	0%	none	Not applicable
Complications	Not applicable	Not applicable	Not applicable

Not applicable: the assessment is not possible because of the insufficient number of included studies

#### Publication bias

**Fig. 3:**
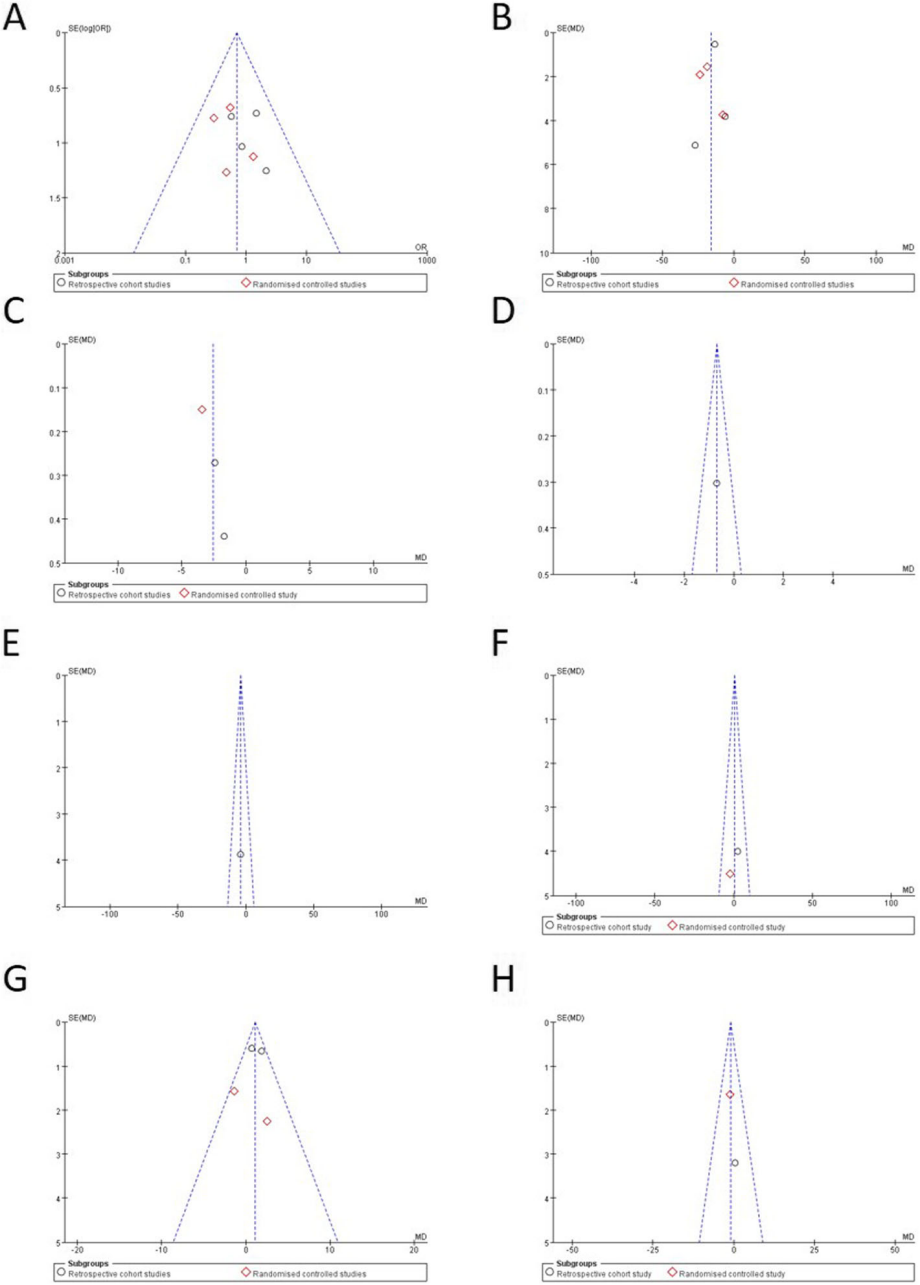
Funnel plots of outcomes. Publication bias assessment. Funnel plots of outcomes, Egger’s linear regression test was used to evaluate asymmetry, and P < 0.05 was set as the level of significance. (**a**) Graft success rate, *p* = 0.288; (**b**) Operation time, p = 0.016; (**c**) Immediate postoperative pain, p = 0.178; (**d**) Pain one day after surgery, insufficient observations for Egger’s test; (**e**) Air conduction, insufficient observations for Egger’s test; (**f**) Bone conduction, insufficient observations for Egger’s test; (**g**) Air-bone gap, *p* = 0.522; (**h**) Air-bone gap reduction, insufficient observations for egger’s test

Publication bias was assessed by funnel plots and Egger’s test. Funnel plots are used to examine whether the results of a meta-analysis may have been affected by publication or other types of bias, and in the absence of bias the plot will resemble a symmetrical inverted funnel. Egger’s linear regression test was used to evaluate asymmetry. According to egger’s test, there is publication bias in the outcomes of graft success rate and average ABG. Further details of the publication bias assessment reported in Fig. 3 and Table 5.

### Effects of interventions

#### Graft success rate

**Fig. 4:**
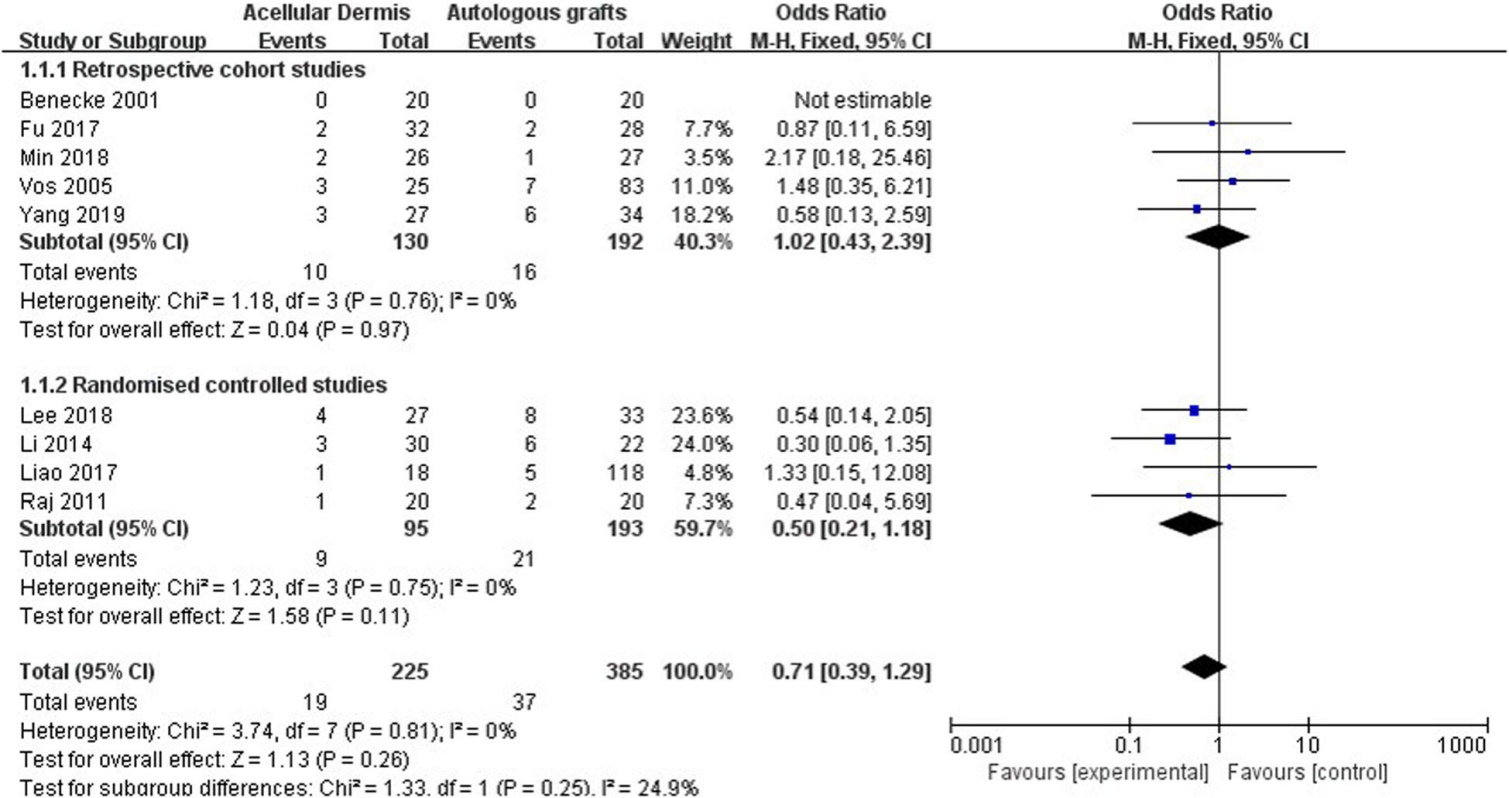
Forest plot of graft success rate

All of the included studies reported the graft success rate, with a total of 610 participants [8, 22–29]. A forest plot of the graft success rate is shown in Fig. 4. No significant difference in graft success rate was found between the ADM group and autologous graft group (OR: 0.71 [0.39, 1.29], *p* = 0.26). The subgroup analysis showed no significant difference between the retrospective cohort studies and randomized controlled studies (*p* = 0.25).

#### Surgery time

**Fig. 5:**
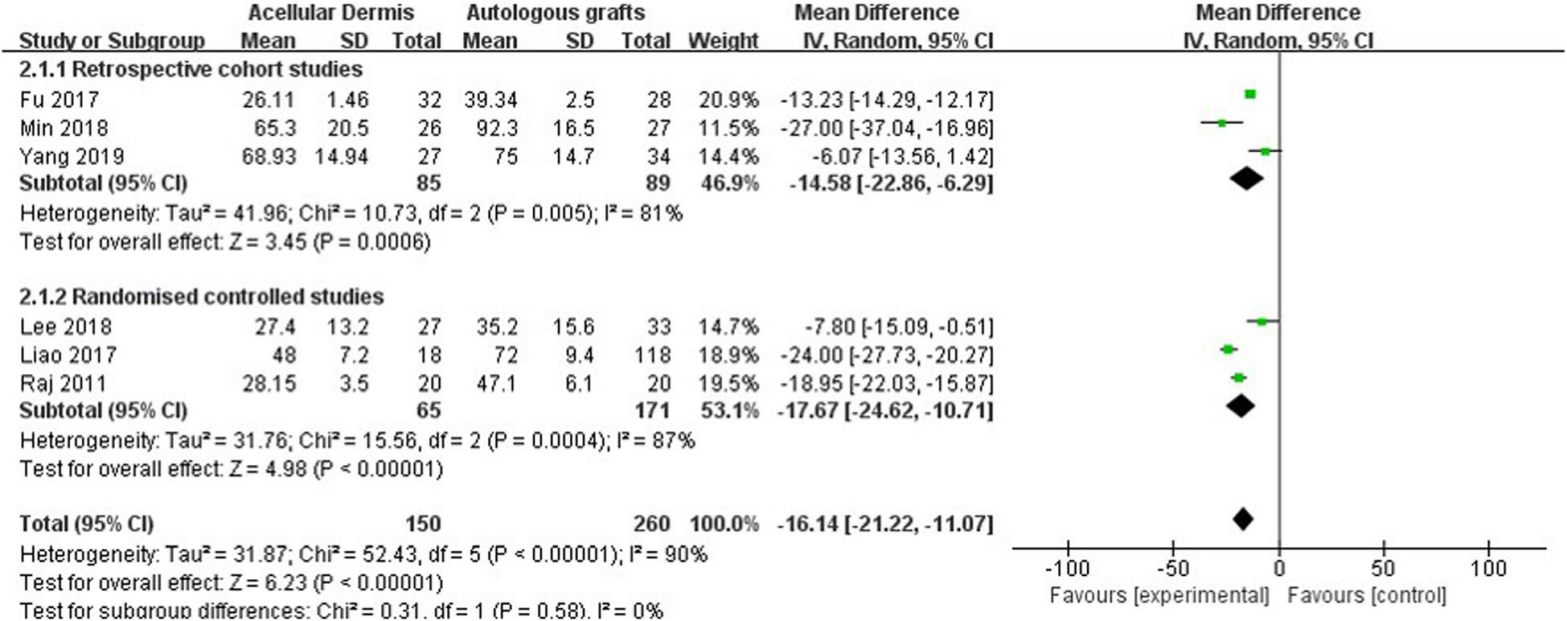
Forest plot of operation time

Seven studies reported the surgery time [22, 24–29]. However, one of these studies only reported the average surgery time and did not report the SD [27]. Therefore, the outcomes of six studies were pooled, with a total of 435 participants. The forest plot of surgery time is shown in Fig. 5. A significant reduction of surgery time was found in the ADM group compared with the autologous graft group (MD: − 16.14 [− 21.22, − 11.07], *p* < 0.00001). The subgroup analysis showed no significant difference between the two groups (*p* = 0.58).

#### Postoperative pain

**Fig. 6:**
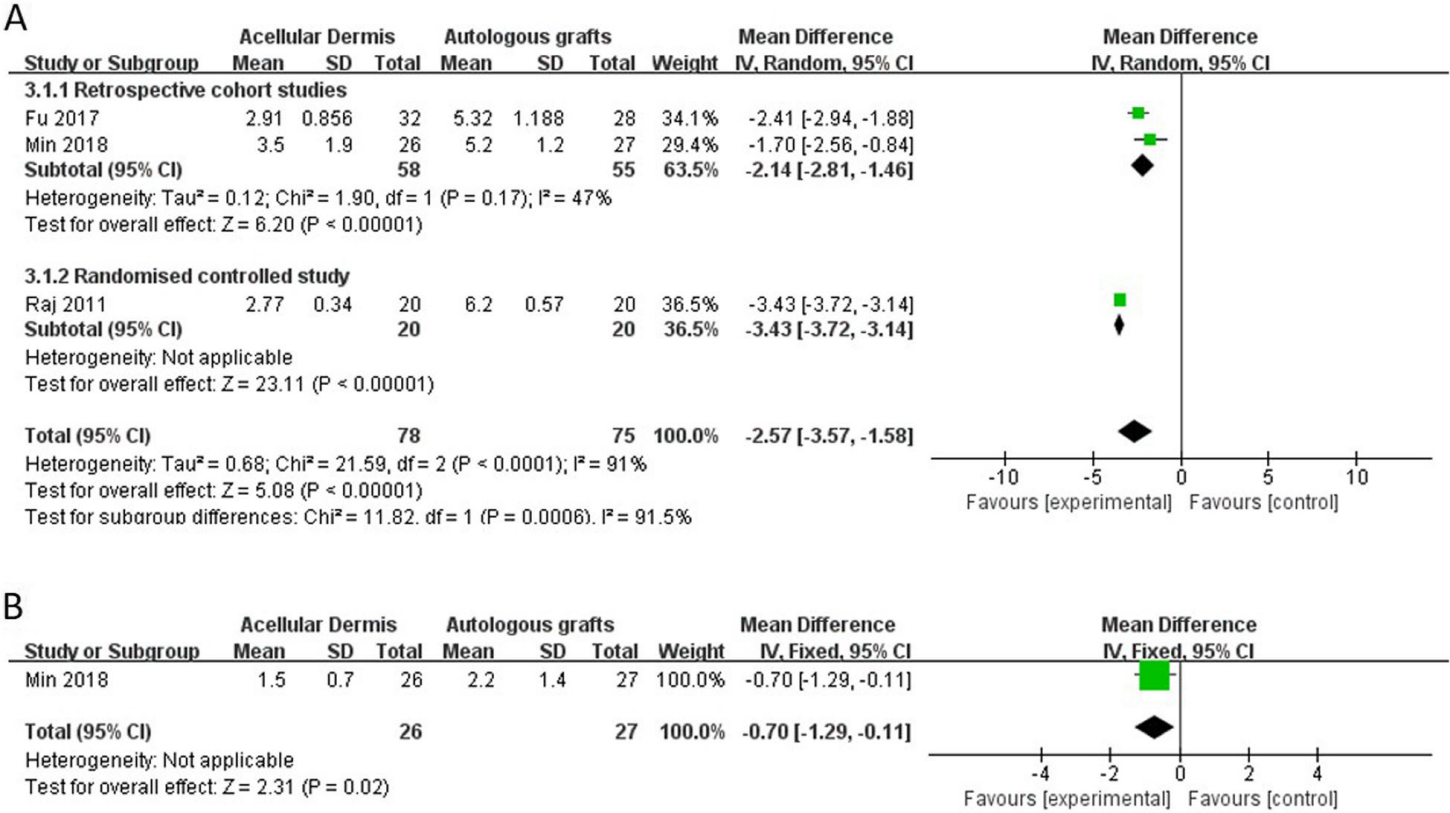
Forest plot of postoperative pain

Three studies reported postoperative pain [24, 26, 29]. The forest plot of postoperative pain is shown in Fig. 6. Two studies reported immediate postoperative pain, indicated by VAS pain scores [24, 29]. One study reported pain scores both immediately postsurgery and 1 day postsurgery [26]. All three studies used type 1 tympanoplasty and administered local anesthesia. Two studies used the temporalis fascia for autologous grafts, and one study used the tragal perichondrium for autologous grafts. A significant reduction of postoperative pain was found in the ADM group compared with the autologous graft group (MD: − 2.57 [− 3.57, − 1.58], *p* < 0.00001; Fig. 6a). Only one study reported pain levels 1 day postsurgery, with a significant reduction of VAS pain scores in the ADM group (MD: − 0.70 [− 1.29, − 0.11], *p* = 0.02; Fig. 6b).

#### Hearing results

**Fig. 7:**
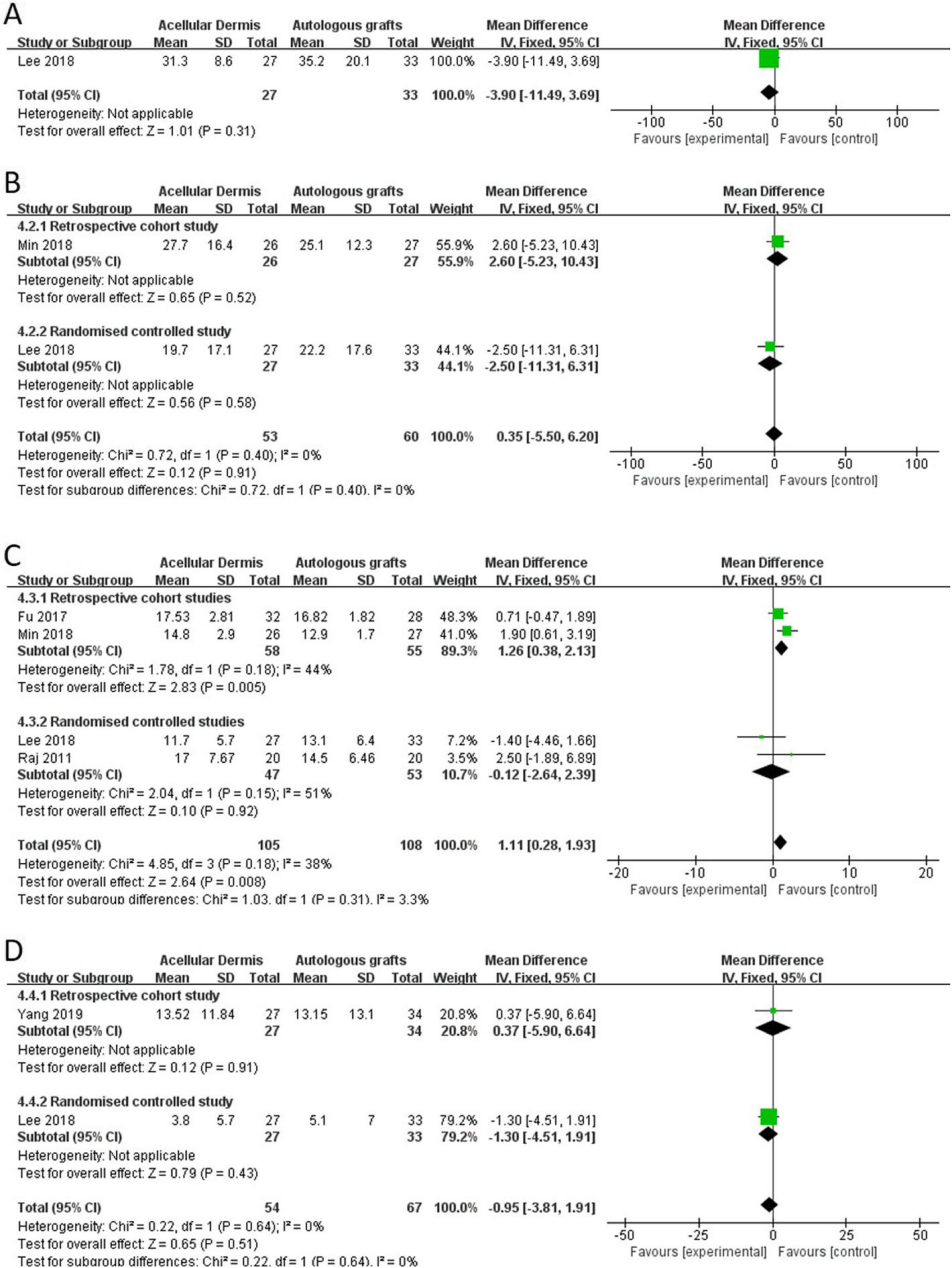
Forest plot of hearing results

The forest plot of the hearing results is shown in Fig. 7. Based on our inclusion and exclusion criteria**,** all of the studies in which audiologic data were collected involved type 1 tympanoplasties and no ossicular chain reconstructions. One study reported AC thresholds [22]. No significant difference in AC thresholds was found between the ADM group and autologous graft group (MD: 0.39 [− 11.49, 3.69], *p* = 0.52; Fig. 7a). Two studies reported BC thresholds [22, 26]. No significant difference in BC thresholds was found between the ADM group and autologous graft group (MD: 0.35 [− 5.50, 6.20], *p* = 0.91; Fig. 7b). Seven studies reported the ABG [22–26, 28, 29]. Three studies reported the average ABG as means and SDs [24, 26, 29], One study reported a reduction of the ABG [28], and one study reported both the average ABG and reduction of the ABG [22]. The other two studies reported the ABG as grading data [23, 25]. Therefore, five studies were included in this analysis. The average ABG significantly increased in the ADM group compared with the autologous graft group (MD: 1.11 [0.28, 1.93], *p* = 0.008). The subgroup analysis of retrospective cohort studies showed a more significant reduction of the ABG in the ADM group (MD: 1.26 [0.38, 2.15], *p* = 0.005). However, a contradictory result from the randomized controlled studies indicated no significant difference in the average ABG between the ADM group and autologous graft group (MD: − 0.12 [− 2.64, 2.39], *p* = 0.92; Fig. 7c). No significant difference in the ABG reduction was found between the ADM group and autologous graft group (MD: − 0.59 [− 3.81, 1.19], *p* = 0.51; Fig. 7d).

#### Complications

**Fig. 8:**
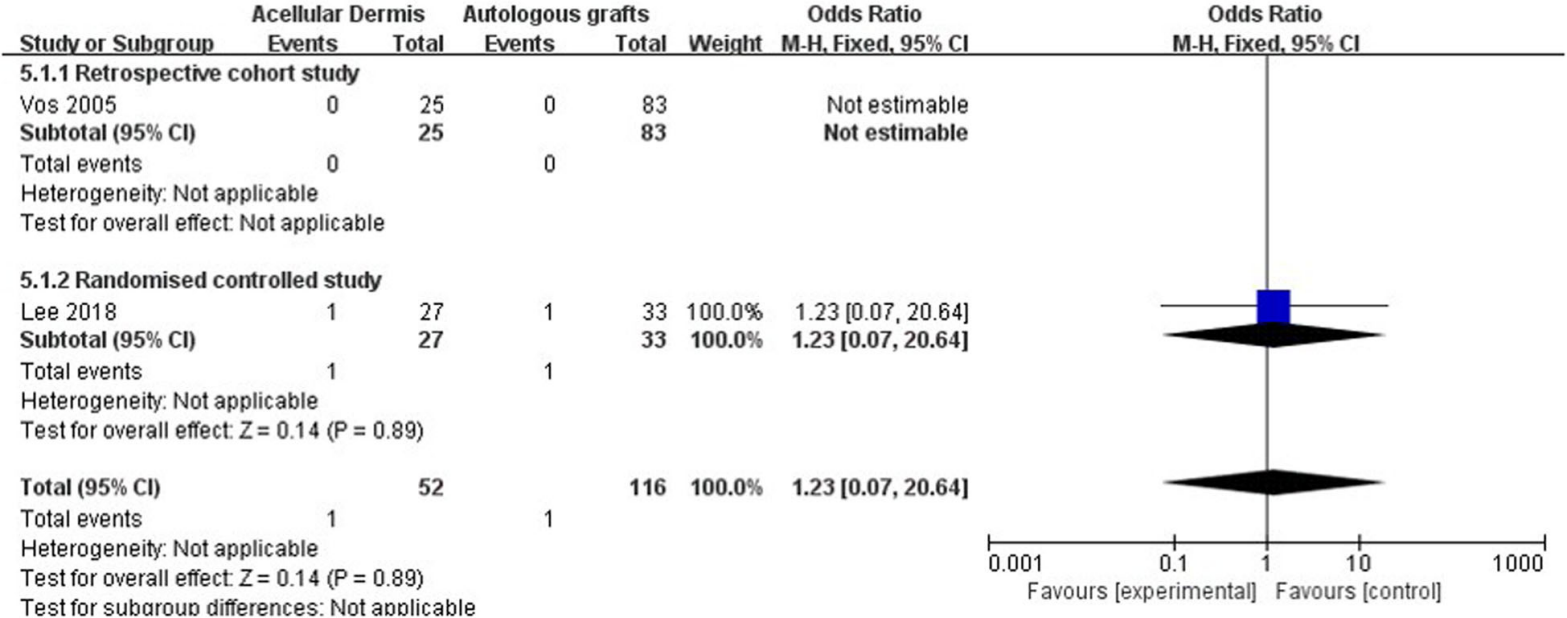
Forest plot of complications

Two studies reported postoperative complications [22, 27]. The forest plot of complications is shown in Fig. 8. No significant difference in the rate of complications was found between the ADM group and autologous graft group (OR: 1.23 [0.07, 20.64], *p* = 0.89).

## Discussion

The present systematic review and meta-analysis included nine studies with a total of 610 participants. We found that compared with autologous grafts, ADM was an effective alternative as a material for TM grafting, with a similar graft success rate and postoperative hearing results. Furthermore, ADM may also significantly reduce surgery time and postoperative pain.

Although no significant difference in the graft success rate was found between the ADM group and autologous graft group, the success rate in retrospective cohort studies (OR: 1.02 [0.43, 2.39]) was slightly higher than in randomized controlled studies (OR: 0.50 [0.21, 1.18]). We investigated the source of this difference, but it did not appear to derive from participant or surgery factors, such as age, type of graft, and surgical technique. We also explored the impact of the size and location of TM perforations on the graft success rate, but the included studies rarely reported relevant information about perforations. One study showed that graft success rates were not significantly different with regard to size or location of the perforations [22]. Another study reported that the graft success rate was higher for small perforations [28]. Because of the limited patient information that was available in the studies, appropriate case selection for the use of ADM is unclear. Additionally, compared with randomized controlled studies, retrospective cohort studies may have potential case selection bias and thus may be a reason why retrospective cohort studies have a slightly higher graft success rate than randomized controlled studies.

A significant reduction of surgery time was found in the ADM group compared with the autologous graft group. Although a high risk of heterogeneity was found among the included studies, this heterogeneity did not appear to be attributable to any one study. All of the studies reported that the use of ADM can significantly shorten tympanoplasty surgery time. Using autologous grafts as material for tympanoplasty usually requires harvesting grafts from tissue first. Compared with autologous grafts, ADM is advantageous because of its direct use and relatively straightforward surgery. Procedures for harvesting and preparing grafts are unnecessary when using ADM, thus shortening surgery times.

Compared with the autologous graft group, postoperative pain was significantly lower in the ADM group. Patients who receive autologous grafts to repair TM perforations require additional incisions. The use of ADM can avoid external skin incisions and soft tissue dissection compared with autologous grafts. The reduction of pain may result from the absence of these incisions. Immediate postoperative pain can be significantly influenced by the specific surgical approach, the use of local anesthesia, and medications that are administered during recovery before self-reporting pain on VASs. However, although the three studies that were included in the analysis all reported the application of local anesthesia before surgery, these studies did not provide information about the surgical approach or medications.

When comparatively evaluating auditory function, differences between AC thresholds, BC thresholds, and the ABG are usually measured before and after surgery. In the present study, the average ABG significantly increased in the ADM group compared with the autologous graft group. However, this increase appeared to derive from bias in one of the studies [26]. When data from this study were excluded, the total MD decreased from 1.11 (0.28, 1.93, *p* = 0.08) to 0.56 (− 0.51, 1.63, *p* = 0.31), the I^2^ decreased from 38% (*p* = 0.18) to 16% (*p* = 0.30), and the overall effect decreased from 2.64 (*p* = 0.008) to 1.02 (*p* = 0.31). The ABG reduction analysis, which did not exclude this study, indicated no significant difference in ABG changes between the ADM group and autologous graft group. Additionally, the ABG value can vary not only according to a decrease in the mere sound conduction component by reducing the AC threshold but also according to changes in the BC threshold before and after surgery [30–32]. However, only one study reported both the BC threshold and ABG [26]. One study reported the AC threshold, BC threshold, and ABG [22]. The frequencies that were tested for pure-tone audiography were also not consistent among the included studies.

Only two studies reported postoperative complications [22, 27], and only two cases of myringitis were reported in one of these studies [22]. No significant difference in complications was found between the ADM group and autologous graft group.

### Limitations

The present meta-analysis has limitations, such as the heterogeneity of some outcomes, and the studies did not distinguish pure-tone audiography data at different frequencies. The main limitation, however, was the high risk of bias of the included studies, especially for randomized controlled studies. More adequately designed clinical trials should be performed in the future to generate solid evidence that may be useful for both clinicians and patients. In addition to the clinical and audiological outcome parameters, further studies should also consider patient-related aspects, such as health-related quality of life, after tympanoplasty [33, 34].

## Conclusions

The present systematic review and meta-analysis confirmed that ADM might be an effective alternative to autologous grafts for tympanoplasty. The use of ADM appears to achieve similar graft success rates and postoperative hearing results, with shortened surgery times and less pain. However, some of the included studies had rather low methodological quality, and more adequately designed clinical trials should be performed in the future.

## Supplementary Information

**Additional file 1.**

## Data Availability

Not applicable.

## Abbreviations

ADM: Acellular dermal matrix
TM: Tympanic membrane
MD: Mean difference
CI: Confidence intervals
OR: Odds risk
VAS: Visual analogue scale
AC: Air conduction
BC: Bone conduction
ABG: Air-bone gap
HRQoL: Health-related quality of life
