# Supplementary material for: Human-derived acellular dermal matrix may be an alternative to autologous grafts in tympanic membrane reconstruction: systematic review and meta-analysis
Source: J Otolaryngol Head Neck Surg. 2021 Jul 6;50:43. doi: 10.1186/s40463-021-00518-w (PMC8261972; doi:10.1186/s40463-021-00518-w)
Supplement: Supplementary file 1 — Additional file 1. [file 40463_2021_518_MOESM1_ESM.docx]

**PubMed**

(((((Acellular Dermis[Title/Abstract]) OR (Acellular Dermal Tissue[Title/Abstract])) OR (Acellular Dermal Graft Tissue[Title/Abstract])) OR (Decellularized Dermal Scaffold[Title/Abstract])) OR (Acellular Dermal Matrix[Title/Abstract])) AND ((Tympanoplasty[Title/Abstract]) OR (Tympanoplasties[Title/Abstract]))

**EMBASE**

('Acellular Dermis':ab,ti OR 'Acellular Dermal Tissue':ab,ti OR 'Acellular Dermal Graft Tissue':ab,ti OR 'Decellularized Dermal Scaffold':ab,ti OR 'Acellular Dermal Matrix':ab,ti) AND ('Tympanoplasty':ab,ti OR 'Tympanoplasties':ab,ti)

**Cochrane library**

((Acellular Dermis):ti,ab OR (Acellular Dermal Tissue):ti,ab OR (Acellular Dermal Graft Tissue):ti,ab OR (Decellularized Dermal Scaffold):ti,ab OR (Acellular Dermal Matrix):ti,ab) AND ((Tympanoplasty):ti,ab OR (Tympanoplasties):ti,ab)

**Ebsco**

(AB, TI Acellular Dermis OR AB, TI Acellular Dermal Tissue OR AB, TI Acellular Dermal Graft Tissue OR AB, TI Decellularized Dermal Scaffold OR AB, TI Acellular Dermal Matrix) AND (AB, TI Tympanoplasty OR AB, TI Tympanoplasties)

**Ovid**

((Acellular Dermis OR Acellular Dermal Tissue OR Acellular Dermal Graft Tissue OR Decellularized Dermal Scaffold OR Acellular Dermal Matrix).ab,ti) AND ((Tympanoplasty OR Tympanoplasties).ab,ti)

**Scopus**

((TITLE-ABS ( 'Acellular Dermis' ) )  OR (TITLE-ABS ( 'Acellular Dermal Tissue' ) )  OR (TITLE-ABS ( 'Acellular Dermal Graft Tissue' ) )  OR (TITLE-ABS ( 'Decellularized Dermal Scaffold' ) )  OR (TITLE-ABS ( 'Acellular Dermal Matrix' ) )) AND ((TITLE-ABS ( 'Tympanoplasty' ) )  OR (TITLE-ABS ( 'Tympanoplasties' ) ))

**Web of science since**

((TI= Acellular Dermis OR AB= Acellular Dermis) OR (TI= Acellular Dermal Tissue OR AB= Acellular Dermal Tissue) OR (TI= Acellular Dermal Graft Tissue OR AB= Acellular Dermal Graft Tissue) OR (TI= Decellularized Dermal Scaffold OR AB= Decellularized Dermal Scaffold) OR (TI= Acellular Dermal Matrix OR AB= Acellular Dermal Matrix)) AND ((TI= Tympanoplasty OR AB= Tympanoplasty) OR (TI= Tympanoplasties OR AB= Tympanoplasties))
